# Correlation between follicular fluid 25-OH vitamin D and assisted reproductive outcomes

**Published:** 2015-06

**Authors:** Laya Farzadi, Homa Khayatzadeh Bidgoli, Morteza Ghojazadeh, Zahra Bahrami, Amir Fattahi, Zeinab Latifi, Vahideh Shahnazi, Mohammad Nouri

**Affiliations:** 1 *Women’s Reproductive Health Research Center, Tabriz University of Medical Sciences, Tabriz, Iran.*; 2 *Liver and Gastrointestinal Disease Research Center, Tabriz University of Medical Sciences, Tabriz, Iran.*; 3 *Department of Clinical Biochemistry, School of Medicine, Tabriz University of Medical Sciences, Tabriz, Iran.*

**Keywords:** *Embryo implantation*, *Vitamin D*, *In vitro fertilization*, *Outcome*

## Abstract

**Background:**

Vitamin D in complex with its receptor by regulating gene expression, endometrium immune response and stimulation of endometrium decidualization can be involved in implantation. So, it seems that the amount of vitamin D in follicular fluids (FF) may have an association with ART success.

**Objective:**

First, we intended to investigate the possible association between levels of follicular fluids 25-OH vitamin D with assisted reproductive outcomes. Second, we examined relationship between 25-OH vitamin D levels with number and quality of oocytes.

**Materials and Methods:**

In a prospective study, 80 infertile female candidates for IVF/ICSI were enrolled. Blood samples (on the day of human chorionic gonadotropin administration) and follicular fluids were taken, and then levels of serum estradiol and follicular fluids 25-OH vitamin D were measured. Also clinical characteristics of patients (duration of infertility, causes of infertility, menstrual status), number and quality of oocytes, number of fertilized oocytes, estradiol levels, and clinical pregnancy were evaluated.

**Results:**

Concentration of FF 25-OH vitamin D in pregnant women was significantly higher than non-pregnant women (p=0.007) but there were no significant differences in age, body mass index (BMI), duration of infertility, menstrual status, number of oocytes, oocytes quality, number of fertilized oocytes, and serum estradiol levels between the two groups. Statistically positive correlation was found between 25-OH vitamin D levels with patient age and implantation rate (r=0.264, p=0.018 and r=0.301, p=0.007 respectively).

**Conclusion:**

The obtained results suggest that vitamin D without affecting the number and quality of oocytes can independently improve implantation rate and IVF outcome.

## Introduction

Existence of vitamin D receptors in the female reproductive system including the uterus, endometrium, ovaries and placenta, represents pivotal role of vitamin D in female fertility (1). Further studies have suggested that vitamin D deficiency is associated with some female reproductive abnormalities especially gestational diabetes, endometriosis, PCOS, bacterial vaginosis, premature labor pains and preeclampsia (2-7). Also it has been reported that in mice with knocked out vitamin D receptor (*VDR*) gene, impaired follicle development, gonadal malfunctions, decreased aromatase expression and activity, pregnancy complications, uterine hypoplasia and infertility can be seen (8, 9). This vitamin in complex with its receptor by regulating of *HOXA10* gene expression, endometrium immune response and stimulation of endometrium decidualization can also be involved in implantation (10).

Considering the importance of vitamin D in pregnancy and implantation and according to Estes *et al* report about decreased expression level of vitamin D binding protein in follicular fluid (FF) as a potential biomarker for ART outcome, it seems that the amount of vitamin D in FF can has a significant association with assisted reproductive technology (ART) success (11). In this regard, Ozkan *et al *have documented that high level of vitamin D in FF and serum is related with chance of conceiving following IVF (12). Bodnar and colleagues also have reported that vitamin D deficiency is associated with reduced pregnancy outcome (2). Rudick *et al* in a similar study showed a direct correlation between vitamin D and IVF success in non-Asian patients but not in Asian population (13). While, some studies have mentioned the lack of this association or even inverse correlation (14, 15).

Roles of estradiol on uterine growth and folliculogenesis during IVF are absolutely clear, but its effects on implantation and IVF outcome are not well understood. Some researchers have investigated association between serum estradiol level and pregnancy rates following IVF, but the results are contradictory (16-18). It has been documented that serum estradiol levels on the day of human chorionic gonadotropin (hCG) administration have a direct correlation with the number of oocytes and an inverse relationship with implantation and pregnancy rate, while some have emphasized the absence of such a relation (17-19). 

Recently, in a study on 1712 patients who underwent their first IVF cycle, it was found that over 10% reduction in estradiol levels after hCG administration leads to a 50% decreasing in clinical pregnancy rate (20). Regarding the role of vitamin D in steroidogenesis and increasing expression and activity of aromatase, it is likely that this vitamin exerts its effects on the number and quality of oocytes and the outcome of pregnancy through increasing estradiol production (21).

Because of mixed results about association of vitamin D and estradiol levels with IVF outcome, in this study we intend to investigate the possible association between levels of serum estradiol and FF 25-OH vitamin D with the number and quality of oocytes and rate of clinical pregnancy.

## Materials and methods

In a prospective study, 80 infertile female (age 31±6 years) who were referred to Al Zahra Hospital of Tabriz between February 2009-February 2010 and were candidate for IVF/ICSI were enrolled. Informed consent was obtained from participants according to the Ethical Committee of Tabriz University of Medical Sciences criteria. All of the 25 to 37 years old patients who were in first ART cycle with one of the following causes of infertility were enrolled in the study; male factor infertility, ovulatory dysfunction, fallopian tube obstruction, and idiopathic. Subjects with history of inflammatory disease, endocrine disorders (hyper- or hypothyroidism, Cushing’s syndrome, hyperprolactinemia, etc.), liver, kidney, or heart disease, immune system diseases, endometriosis, consuming drugs interfering with vitamin D metabolism and previous ART cycle experience were excluded.

In all patients, long protocol with administration of exogenous gonadotropin (Gonal-F, Merck, Germany and Menogon^®^, Ferring, Germany) was used to stimulate ovulation and when at least 2-3 follicles reached 18 mm, venous blood samples were taken for assessment of serum estradiol and 10,000 IU human chorionic gonadotropin (Choriomon, Lugano, Switzerland) was administered intramuscularly on the same day. Serum was isolated by centrifugation, and for subsequent assay was kept at -70^o^C.Transvaginal-guided follicular puncture and aspiration of FF was performed under general anesthesia 34 hr after administration of HCG.

FF was centrifuged at 3000g for 15 min and the supernatant was stored at -70^o^C for 25-OH vitamin D measuring. Then the number and quality of oocytes and the number of fertilized oocytes and also clinical pregnancy rate in each patient were evaluated. For grading oocyte we used method which explained previously by Xia (22). Briefly oocytes were graded into 4 groups; 1) large perivitelline space with fragmented first polar body, 2) large perivitelline space with intact first polar body, 3) normal perivitelline space with fragmented first polar body and 4) normal perivitelline space with intact first polar body.

The number of fertilized oocytes was counted 84 hr after ICSI and depending on the number of embryos available, a maximum of 3 high-quality embryos (based on cleavage and morphology) were transferred to the uterus. Clinical pregnancy was considered when the transvaginal ultrasound revealed intrauterine gestational sac. Implantation rates were obtained from ratio of visible sacs number to transferred embryos number. In all samples serum estradiol level was measured by commercial ELISA kit (Roche, Germany) and 25-OH vitamin D level in FF was evaluated by enzyme immunoassay method using DIAsource ImmunoAssays^®^ kit (Belgium).

**Statistical analysis**

Data are provided as mean±SD and normal distribution of data was examined by Kolmogorov-Smirnov test. To compare quantitative data between groups, Mann- Whitney U test was used and Chi-square test was employed for qualitative data. Logistic regression was used to determine the regression model. Spearman test was used to evaluate correlation between the study parameters. Data analysis was performed using SPSS V.16 software and statistical significance was assumed at the p≤0.05 level.

## Results

Of 80 patients, in 18 (22.5%) subjects clinical pregnancies were observed, but in 62 (77.5%) cases the pregnancy did not occurred. Basal characteristics of pregnant and non-pregnant patients are shown in table I. Statistical analysis showed no significant differences in age, body mass index (BMI), duration of infertility, menstrual status, between the two groups. As shown in table II, we did not find significant differences in the number of oocytes, oocytes quality, number of fertilized oocytes and serum estradiol levels (day of hCG administration) between pregnant and non-pregnant women. Only a significant difference was found in concentration of FF 25-OH vitamin D between the two groups which the concentration was higher in the group with clinical pregnancy. Evaluating the ratio of appeared sacs number to transferred embryos number in pregnant women demonstrated that the average rate of implantation is 52.3±22.4.

In the assessment of correlation between variables, no significant correlation was observed between FF 25-OH vitamin D levels with BMI, duration of infertility, number of oocytes, number of fertilized oocytes and estradiol levels. Statistically positive correlation was found between 25-OH vitamin D levels with patient age and implantation rate (Table III). Logistic regression analysis revealed that after controlling for the effects of age, for each ng/ml increasing of 25-OH vitamin D level in follicular fluid, the rate of clinical pregnancy following IVF increases 1.2 times.

**Table I:** Basal characteristics of clinical pregnant and non-pregnant patients

**Parameters**		**Clinical pregnant (n=18)**	**Non-Clinical pregnant (n=62)**	**p-value**
Age (year)*^a^		31.9 ± 4.9	29.9 ± 4.8	0.052
BMI (kg/m2)*^a^		27.2 ± 3.8	27.6 ± 3.8	0.954
Duration of infertility (year)*^a^		7.3 ± 4	6.5 ± 3.6	0.513
Causes of infertility**^b^				
	Male factor (n=46)	11 (61.1%)	35 (56.5%)	0.70
	Ovulatory dysfunction (n=13)	2 (11.1%)	11 (17.7%)	
	Fallopian tube obstruction (n=8)	1 (5.6%)	7 (11.3%)	
	Idiopathic (n=13)	4 (22.2%)	9 (14.5%)	
Menstrual status**^b^				
	Regular	16 (88.9%)	53 (85.5%)	0.529
	Irregular	2 (11.1%)	9 (14.5%)	

BMI: Body mass index.

a : Mann-Whitney U

b : Chi-square test

* Data are presented as mean±SD.

** Data are presented as n (%)

**Table II:** Cycle characteristics of clinical pregnant and non-pregnant patients

**Parameters**		**Clinical pregnant (n=18)**	**Non-Clinical pregnant (n=62)**	**p-value**
Number of oocytes*^a^		12.2 ± 4.6	11.4 ± 7.1	0.352
Oocytes quality**^b^				
	G1	0 (0%)	0 (0%)	0.395
	G2	78 (35.45%)	233 (33.28%)	
	G3	128 (58.18%)	350 (50%)	
	G4	14 (6.37%)	117 (16.72%)	
Number of fertilized oocytes*^a^		6.2 ± 3.3	5.3 ± 4.7	0.120
Estradiol (pg/ml)*^a^		2751.4 ± 1224.6	2546.6 ± 1464	0.595
25-OH vitamin D (ng/ml)*^a^		15.8 ± 4.5	11.6 ± 5.5	0.007

G1: large perivitelline space with fragmented first polar body.

G2: large perivitelline space with intact first polar body

G3: normal perivitelline space with fragmented first polar body and

G4: normal perivitelline space with intact first polar body.

* Data are presented as mean±SD.

** Data are presented as n (%)

a : Mann-Whitney U test

b : Chi-square test

**Table III:** Correlation between 25-OH vitamin D levels with other study variables

**Variable**		**rho**	**p-value**
Age		0.264	0.018
BMI		0.054	0.633
Duration of infertility		0.002	0.988
Number of oocytes		0.198	0.078
Number of fertilized oocytes		0.163	0.149
Estradiol levels		0.067	0.560
Implantation rates		0.301	0.007
Oocytes quality			
	G1^a^	-	-
	G2	0.045	0.605
	G3	0.068	0.520
	G4	0.024	0.833

BMI: Body mass index

Spearman test was used to evaluate correlation between 25-OH vitamin D levels with the factors and correlation coefficient (rho) with p-value is presented for each variable.

a Oocyte with G1 quality did not observed

## Discussion

Several studies have investigated association between IVF outcome and different factors in follicular fluid such as anti-mullerian hormone, pregnancy-associated plasma protein-A, cholesteryl ester transfer protein and vitamin D (12, 23-25). Studies have offered that vitamin D could be involved in important functions of female reproductive system such as steroidogenesis, folliculogenesis, implantation and developing fetus (8, 21, 26, 27). It was recently reported that this vitamin could possibly be a prognosis factor for pregnancy success following IVF, although the results are contradictory and the mechanisms of impact on IVF results has not been exactly determined (12).

In this study, we have examined the association between 25-OH vitamin D of FF with the number and quality of oocytes, number of fertilized oocytes and also implantation and clinical pregnancy rates. No significant differences between pregnant and non-pregnant groups were found in age, BMI, duration of infertility, menstrual status, number of oocytes, oocytes quality, number of fertilized oocytes and serum estradiol levels (day of hCG administration), whereas FF levels of 25-OH vitamin D in pregnant group was significantly higher than non-pregnant subjects.

Our results also revealed a positive correlation between 25-OH vitamin D levels with implantation rates and age, while such a relationship were not found with BMI, duration of infertility, number of oocytes, number of fertilized oocytes and serum estradiol levels. The obtained results suggest that vitamin D without affecting the number and quality of oocytes can independently improve implantation. In similar studies, Anifandis, Ozkan and Rudick have observed a direct link between vitamin D levels and IVF outcome (12, 13, 15). Studies have shown that vitamin D deficiency or defect in its receptor can reduce chances of pregnancy by disrupting follicles and oocytes development and gonads function especially in estradiol synthesis, so it has been proposed that maybe high levels of vitamin D through affecting these items can improve pregnancy outcome (6, 8, 9, 12). 

However, our study demonstrated that vitamin D without having effect on the number and quality of oocytes, basal estradiol levels and number of fertilized oocytes can increase chance of pregnancy by 1.2-fold. In some of previous studies association between vitamin D level with number and quality of oocytes and embryos has not been found (14, 28). This indicates that potential impact of vitamin D on implantation rate is probably by binding to its receptors in the endometrium. Ozkan *et al* have also suggested that vitamin D may increase endometrial receptivity and chance of pregnancy via binding to its receptor in the endometrium (12). One of the possible mechanisms of vitamin D-receptor complex can be increasing the expression of *HOXA10* gene which has an essential role in implantation (10). Moreover, it is likely that vitamin D may play a role in uterine receptivity by modulating endometrial immune response, as it has been seen that this vitamin can reduce the decidual T-cell activity and synthesis of cytokines, interleukin 1 and 6 and TNFα which are essential in embryo implantation and uterine receptivity (27, 29).

On the other hand some researchers have documented that there is an inverse correlation between vitamin D levels with the number and quality of follicles and oocytes and also IVF success, although our results did not show such a correlation (15). Anifandis *et al* have reported that vitamin D decreases chance of pregnancy through affecting insulin function and decreasing FF glucose which is essential for oocyte and granulosa development (15). Some studies also have emphasized the lack of association between vitamin D levels with the quality and number of oocytes and pregnancy rate (30).

These conflicting results are probably due to the low sample size, different causes of infertility in women, lack of identical protocol in ovulation induction and eclipse of pregnancy achieving by various factors. The advantages of our study are using similar protocol of treatment for patients, lack of differences in age, BMI, menstrual status, causes of infertility, number and quality of oocytes, number of fertilized oocyte and basal estradiol level between pregnant and non-pregnant patients which cause revealing the effects of vitamin D on implantation and pregnancy success. However the sample size in this study was relatively low, and for obtaining a clear conclusion and understanding underlying mechanisms more investigations with high sample size are needed.

## Conclusion

In conclusion we have found that vitamin D without affecting the number and quality of oocytes can independently improve implantation rate and IVF outcome.
